# Retained surgical sponges, needles and instruments

**DOI:** 10.1308/003588413X13511609957218

**Published:** 2013-03

**Authors:** D Hariharan, DN Lobo

**Affiliations:** Nottingham University Hospitals NHS Trust, Queen’s Medical Centre, Nottingham, UK

**Keywords:** Retained, Sponges, Foreign bodies, Instruments, Counts, Prevention, Surgery

## Abstract

**Introduction:**

Retained sponges and instruments (RSI) due to surgery are a recognised medical ‘never event’ and have catastrophic implications for patients, healthcare professionals and medical care providers. The aim of this review was to elucidate the extent of the problem of RSI and to identify preventative strategies.

**Methods:**

A comprehensive literature search was performed on MEDLINE^®^, Embase™, the Science Citation Index and Google™ Scholar for articles published in English between January 2000 and June 2012. Studies outlining the incidence, risk, management and attempts to prevent RSI following surgical intervention were retrieved.

**Results:**

The overall incidence of RSI is low although its incidence is substantially higher in operations performed on open cavities. Sponges are the most commonly retained item when compared with needles and instruments. Clinical presentation is varied, leading to avoidable morbidity, and the error is indefensible medicolegally. Risk factors include emergency operations, operations involving unexpected change in procedure, raised body mass index, and a failure to perform accurate sponge and instrument counts. The existing strategy for prevention is manual counting of sponges and instruments undertaken by surgical personnel. This, however, is fallible. Computer assisted counting of sponges using barcodes and gauze sponges tagged with a radiofrequency identification device aiding manual counting have been trialled recently, with success.

**Conclusions:**

Vigilance among operating theatre personnel is paramount if RSI is to be prevented. Prospective multicentre trials to assess efficacy of new technologies aiding manual counting should be undertaken if this medical error is to be eliminated completely.

The retention of foreign bodies (sponges, needles and instruments) in a patient after a surgical procedure is a medical error that often results in adverse consequences for patients and can seriously implicate the healthcare personnel involved. This error is included in the list of 27 ‘never events’ released by the National Quality Forum in the US^1^ and also in the guidance issued by the UK Department of Health.^2^

Surgery to the wrong patient, the wrong side, the wrong site and retention of foreign bodies after a surgical procedure are entirely preventable causes of morbidity and, perhaps, mortality.^3^ Medical negligence in the case of retained sponges and instruments (RSI) is proven easily as a result of the doctrine of *res ipsa loquitur* (‘the thing itself speaks’). The fact that an object was left in a space where it does not belong becomes indefensible. Medicolegal and compensation costs associated with RSI are high, even if there has been little or no harm to the patient. Costs vary from $37,041 to $2,350,000 (approximately £23,000 to £1,460,000) per incident, with an average cost per case estimated at $95,000 (≈£59,000).^4–6^ This review was undertaken to enumerate the extent of the problem of RSI after surgical procedures with the aim of identifying strategies for reducing, if not eradicating, it.

## Methods

A comprehensive literature search was performed on MEDLINE^®^, Embase™, the Science Citation Index and Google™ Scholar for articles published in English between January 2000 and June 2012. Key search words (including retained swabs, sponges, surgical instruments, needles, prevention, risks and surgical counts) were used in combination with the Boolean operators AND, OR and NOT. The search was supplemented using the ‘related article’ function. Bibliographies of selected articles were further searched manually for studies that were missed in the initial electronic search. Older publications were included if relevant.

## Incidence

The incidence of RSI varied from 1 in every 1,000 to 1,500 intra-abdominal operations in studies performed in the early 1980s^7,8^ while more contemporary studies suggest an incidence of 1 in 5,500 to 1 in 18,760 inpatient operations.^3,9,10^ A total of 496 patient safety incidents involving retained sponges and instruments occurring between 1 April 2007 and 31 March 2008 were reported to the UK National Reporting and Learning System.^11^

The reasons for the varied incidence of RSI include: the retrospective nature of studies, a reluctance on the part of hospitals and clinicians to disclose these errors publically due to their sensitive nature, incidental discovery of the RSI after many years as patients may remain asymptomatic, and confidentiality requirements of insurance and legal claims hampering publication of data on RSI.^3,12^

A surgical sponge is the most commonly reported retained item following surgery while reports of retained needles and instruments are extremely rare.^3,9,12,13^ No surgical specialty and no surgical procedure are immune to the problem. Wan *et al* reviewed 254 cases published between 1963 and 2008, and found the abdominal/pelvic cavity/vaginal vault (74%) to be the most common site for RSI, followed by the thoracic cavity (11%).^14^ These findings are similar to those of other studies,^3,9,12,15^ thereby implicating the surgical personnel involved in these operations as the prime offenders.

## Predisposing risk factors

The risk factors leading to RSI after surgery have been elucidated by two observational studies.^3,12^ Gawande *et al* performed a retrospective case-control analysis of all malpractice claims and incident reports involving RSI filed between 1 January 1985 and 1 January 2001 across 22 hospitals in the US.^3^ A total of 60 cases involving 61 RSI events were identified, with 4 hospitals accounting for 83% of cases. For each case of RSI, four patients who had undergone the same procedure as the given patient during the same period were selected as controls. Lincourt *et al* replicated the above study by performing a case-control analysis involving retrospective review of medical records from a single institution over a ten-year period (January 1996 – December 2005).^12^ They cross-referenced the **International Classification of Diseases code for unintentionally retained foreign body during surgery with their billing and reimbursement database, and found 30 cases of RSI.

The risk factors for RSI identified in the two studies^3,12^ are summarised in Table 1. The results obtained from these two studies are discordant mainly because of the low incidence of retained foreign objects along with the methodology used.

**Table 1:** Risk factors for retained foreign objects identified across two retrospective case-control studies

Study	Risk factors for retained swabs and instruments	*p*-value	Risk ratio (95% confidence interval)
Gawande^3^	Operations performed on emergency basis	**<0.001**	8.8 (2.4–31.9)
	Body mass index (per 1 unit increment)	**0.01**	1.1 (1.0–1.2)
	Unexpected change in operation	**0.01**	4.1 (1.4–12.4)
	Multiple surgical teams	0.1	3.4 (0.8–14.1)
	Female sex	0.13	0.4 (0.1–1.3)
	Estimated blood loss (per 100ml increment)	0.19	1.0 (1.0–1.0)
	Change in nursing staff during procedure	0.24	1.9 (0.7–5.4)
	Counts of sponges and instruments	0.76	0.6 (0.0–13.9)
Lincourt^12^	Number of major procedures performed	**0.008**	1.6 (1.1–2.3)
	Incorrect counts recorded	**0.02**	16.2 (1.3–197.8)
	Multiple surgical teams	>0.05	5.4 (0.9–33.1)
	Unexpected change in operation	>0.05	Not mentioned
	Operation theatre time	>0.05	Not mentioned
	Procedures performed after 5pm	>0.05	Not mentioned
	Emergency procedures	>0.05	Not mentioned

## Clinical presentation and management

**Figure 1:**
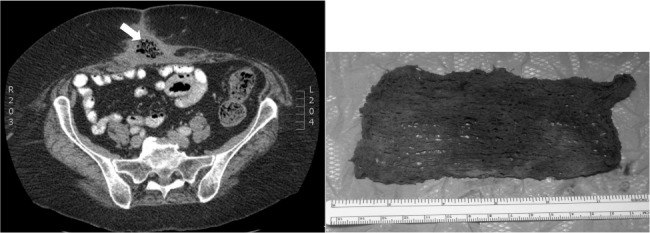
An obese patient developed a wound infection at the site of an incisional hernia repair performed 10 weeks previously. This was treated in the community with negative pressure wound therapy (NPWT). Foul-smelling discharging pus persisted from a wound sinus after cessation of NPWT. Computed tomography (left) showed an area of inflammation with multiple air pockets (arrow) in the subcutaneous tissue of the anterior abdominal wall. Wound exploration under general anaesthesia revealed a sponge (without a radiopaque marker) used for the NPWT dressings in the subcutaneous fat (right). The sponge was removed, the wound healed by secondary intention and the patient made an uneventful recovery.

The clinical presentation of RSI is varied, and depends on the site and type of tissue reaction elicited, as shown in Table 2.^3,8,16,17^ Retained surgical sponges may elicit either an exudative or an aseptic fibrous type of tissue reaction.^18^ The exudative kind of reaction presents fairly early in the postoperative period, causing infections secondary to bacterial contamination. The aseptic fibrous type of reaction is slow in comparison; it involves fibroblasts leading to formation of adhesions, granulomas or pseudotumours, with patients remaining asymptomatic for many years.^19^ Imaging is the key to diagnosis. Computed tomography is the modality of choice to exclude RSI (Fig 1). Plain radiography is also used commonly although it has a false negative rate of 10–25% despite the presence of radiopaque markers on surgical sponges.^20^ Magnetic resonance imaging and other relevant radiological techniques such as barium contrast studies may also be used depending on the clinical situation.^17^

**Table 2:** Clinical presentation of retained sponges and instruments

ASYMPTOMATIC
Detection is incidental
**SYMPTOMATIC**
*Early*
Unexplained pain, features of generalised sepsis, formation of abscess
*Delayed*
Non-healing wounds, discharging sinuses, mass, signs and symptoms of intestinal obstruction, internal fistulisation, transmural migration and spontaneous expulsion

Retained objects can cause multiple problems (Table 3), and treatment involves surgical removal of the items once diagnosed, even if the patient is asymptomatic. The time interval between initial surgery, diagnosis and removal of RSI is clinically relevant as the morbidity and mortality is much less if the RSI is removed immediately after the offending operation than with delayed diagnosis and retrieval.^19^ Needles are less likely to cause serious clinical consequences and are no different from retained metal shrapnel. The evidence for benefits of removal of these items is unclear and needs to be evaluated on a case-by-case basis, weighing up the risks and benefits of performing another operation.^21^ In any case, a frank discussion must be had with the patient and appropriate measures must be taken for reporting the incident according to local policy.

**Table 3:** Outcomes of patients with reported retained sponges and instruments (*n*=90) across two retrospective case-control studies^3,12^

Outcomes	Number of cases
Death	1
Readmission to hospital	40
Reoperation	62
Intra-abdominal abscess or sepsis	26
Small bowel obstruction / intestinal fistulation	10
Visceral perforation	5

## Value of counting

Counting of items before and after use, at the time of surgery, is the most widely used method for the screening of RSI, with established protocols in place.^22,23^ Egorova *et al* retrospectively analysed count discrepancy data stored in a web-based ‘near miss’ and patient harm reporting system over a four-year period (2000–2004).^10^ Of the 153,263 operations checked, they estimated count discrepancies to occur in 1 in 145 operations (0.69%). The test characteristics of surgical counting as a screening method were determined (sensitivity 77.27%, specificity 99.32%, positive predictive value (PPV) 1.6%, negative predictive value 100%). The low PPV was a result of the low frequency of RSI events (17 true positives, 5 false negatives). The odds of a foreign body being left behind in a patient increased by >100 times when the count was discrepant (positive likelihood ratio 113.3).

Greenberg *et al* designed a prospective observational field study to estimate the rate of intraoperative count discrepancies.^24^ They sought to determine how these discrepancies translate into meaningful problems (misplaced items or RSI), and to examine the relationship between personnel changes and count discrepancies. They observed a total of 2,476 distinct counting episodes during 148 cases (16.6 counting episodes per case) with 8.6 minutes taken for each count to be performed. They found 29 count discrepancies occurring in 19 cases. The majority of discrepancies again involved sponges (45%), followed by instruments (31%) and needles (21%). Of these 29 discrepancies, 59% were due to misplaced items, representing potential RSI, while 41% were due to human error in arithmetic. The analysed causes of count discrepancies across the above two studies are summarised in Table 4, showing that mere counting of sponges and surgical instruments undertaken by allied surgical staff in theatre is prone to error.^10,24^

**Table 4:** Significant predictors of surgical count discrepancy when count performed in the operating theatre at the time of surgery

Study	Predictors of count discrepancy
Egorova^10^	Increased duration of surgery (every additional 2 hours increased the probability of discrepancy by a factor of 2.67) Presence of multiple nursing teams in theatre (80% of count discrepancies arose when more than 2 nursing teams participated) Surgical procedures performed late in the day excluding procedures performed as emergency or on weekends or holidays
Greenberg^24^	Changeover of surgical personnel in operating theatre while procedure being performed (count discrepancies were 3 times more likely when personnel change involved either the surgical technologist or circulating staff)

## Need for preventive strategies

Counting as a screening strategy for RSI is not foolproof. Most studies advocate standardisation, development of local counting protocols, and adherence to counting sponges and instruments preoperatively as well as multiple times intraoperatively.^3,9,12^ These protocols are labour intensive and can occupy up to 14% of operative time.^25^ This should be followed by detailed inspection by the surgeon of the body cavity, ruling out RSI, prior to closure of the body cavity. Count discrepancies should automatically universally trigger a prompt and thorough search for the item, and if the discrepancy persists, appropriate imaging (radiography/computed tomography) should be performed to look for retained objects.

In 2003 Minnesota became the first state in the US to promulgate legislation requiring ‘never events’ to be disclosed to the public.^26^ Cima *et al* from the Mayo Clinic in Rochester, Minnesota, a high volume tertiary referral facility, audited their experience of RSI retrospectively from 2003 to 2006.^9^ They instituted postoperative high-resolution x-ray screening of patients undergoing operations involving body cavities as a routine preventive strategy in a dedicated suite to rule out retained objects before entering the recovery room. A total of 191,168 operations were performed and 68 RSI related events reported; 34 of these were classified as near-miss events, mostly due to incorrect surgical counts, where postoperative x-ray ruled out RSI.

The other 34 cases in the study by Cima *et al* were true RSI, of which 20 (59%) were found due to routine postoperative high resolution surveillance x-rays.^9^ In all of these 20 cases, the surgical counts performed were reported correct at the time of completion of the case. In the 14 remaining cases where counts were incorrect, intraoperative x-rays were done in 4 cases that confirmed RSI. In six cases, x-rays were not performed (in two cases the RSI was coughed up spontaneously, in two cases sponges were retained in the vaginal vault, in one case the retained sponge was retrieved before x-rays were performed and in the final case it was decided against looking for the RSI given the poor clinical condition of the patient). In four cases, RSI were missed despite routine postoperative x-ray screening.

In Mayo Clinic study, therefore, the RSI was discovered within 24 hours of the offending operation in 20 of 34 cases (59%).^9^ This was primarily due to routine postoperative x-ray surveillance. Immediate retrieval was possible in 27 cases (21 needed an additional surgical procedure and in 6 cases the RSI was removed without an operation). In the remaining seven cases, it was decided not to attempt removal in six cases while in one case the attempted operative removal failed.

Cima *et al*’s analysis found no significant association of RSI with risks factors previously identified by Gawande *et al* ^3^ and Lincourt *et al* ^12^ but **confirmed sponges to be most frequently counted during operations as well as the most frequently missed item.^9^ They believed that the complex environment of the operating room, multiple distractions, competing tasks and poor communication among the members of the surgical team hampered the sponge and instrument counting process, leading to RSI.

Concerned as an organisation and aiming to reduce RSI incidents to zero, the Mayo Clinic instituted a multidisciplinary, multiphase managerial approach in 2005.^27^ They implemented key initiatives (performing defect analysis and policy review), initiated awareness, enhanced communication, and formed a rapid response events leadership team to monitor and control any RSI event. The effect of these initiatives was audited, revealing RSI events to occur once every 69 days compared with once every 16 days previously. They believed that the adoption of preventative strategies such as postoperative x-ray surveillance along with management driven initiatives to change clinical practice were key patient safety processes leading to the reduction of RSI.

Despite the steps taken, as an institution, they were still far away from achieveing their goal of reducing RSI rates to zero. There was a realisation that human efforts of counting sponges needed to be complemented. There was an urgent need to incorporate and evaluate new technologies that could be used to assist existing methods to reduce rates of retained objects.

## Advances in sponge counting

A prospective, blinded, experimental clinical trial was conducted for the first time at Stanford University using gauze sponges tagged with a radiofrequency identification (RFID) microchip and a handheld scanning device.^28^ A total of 8 patients were enrolled and 28 RFID tagged sponges were placed in them. The handheld device detected all sponges correctly with no false positive or negative results. These results were extremely encouraging. However, some concerns were raised regarding human errors in performing an incorrect scan, the timing of the scan possibly also impacting on the final result and the lack of cost–benefit analyses limiting the full implementation of such technology.

In a separate study, traditional counting protocols were compared with computer assisted counting of sponges using barcodes in a randomised control trial involving 298 patients.^29^ The barcode system detected significantly more counting discrepancies than the traditional system, which included misplaced and miscounted sponges. This study highlighted the increased frequency with which count discrepancies go unnoticed when traditional counting methods are applied.

In an effort to completely eliminate RSI, the Mayo Clinic evaluated a data matrix coded sponge (DMS) counting system in addition to standard manual counting protocols.^30^ Similar to the barcode system, it included individual unique data matrix coded sponges along with a data matrix scanner, which kept a running count of every sponge introduced and removed from the sterile field. Two internal trials were conducted to assess suitability and, on satisfactory performance in the trials, this technology was fully implemented. Safety performance (incidence of retained sponge products and staff satisfaction) was measured, incorporating DMS to the previously existing RSI risk reduction strategy.

Implementation across the institution was made possible after a two-month period of staff education and training. In the 18-month period following the implementation of the new RSI risk reduction strategy, not a single sponge was reported retained, with a negligible increase in overall operating times, a short learning curve and good acceptance among staff members. This was a considerable feat for an organisation that was averaging a retained sponge every 16 days prior to 2003.^27^ Thus, through focused, concerted and coordinated efforts, the Mayo Clinic, Rochester, as an institution, implemented multiple preventive strategies and audited its results serially to show a dramatic improvement and reduction of RSI rates.

**Figure 2:**
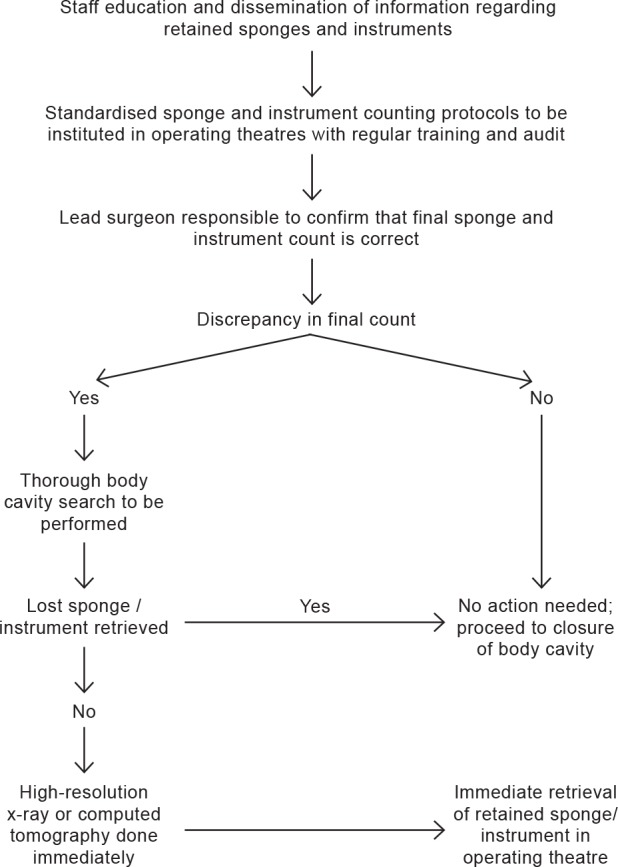
Suggested algorithm to prevent retained sponges and instruments

Each preventive intervention strategy (surgical counting, postoperative x-ray screening) along with new mentioned technologies has their advantages and disadvantages (Table 5). The operating theatre environment is complex, dynamic and at times stressful, and, consequently, human errors are easily missed. The evidence to support the routine use of the new technologies assisting in manual counting of sponges is currently scant. All major surgical institutions must therefore endeavour to analyse their individual operating theatre practices and put in place standard operating protocols to prevent RSI. An algorithm based on the available evidence for the prevention of RSI is shown in Figure 2.

**Table 5:** Advantages and disadvantages of strategies to prevent retained sponges and instruments

Preventive strategy	Advantages	Disadvantages
Counting of sponges and instruments^10^	Standard procedures and protocols in place	Labour intensive Error prone
Intraoperative x-ray screening^10^	Negligible clinical harm Easy to deploy	Poor quality films Poor yield
Routine postoperative high-resolution x-ray surveillance^9^	Negligible clinical harm	High set up costs Logistics False negative rate 10–25% Unnecessary radiation exposure
Sponges tagged with radiofrequency identification chip^28^	High detection accuracy under test conditions	Efficacy unproven No RCTs yet Prone to errors
Barcoded or data matrix coded sponges as adjunct to existing counting protocols^29,30^	Technology in use in medicine Improved detection of miscounted / misplaced sponges in RCT Strategy implemented in a single institution with positive results	Increase in time to count sponges Learning curve to adapt to new technology Cost vs benefit needs to be determined appropriately

RCT = randomised controlled trial

## Conclusions

RSI is an undesirable and preventable cause of surgical morbidity and mortality. As responsible healthcare professionals, we must remain vigilant at all times to the threat posed by RSI. Adoption of new technologies and working alongside industry must be encouraged to assist existing methods of counting processes and protocols. Furthermore, prospective multicentre studies to assess the efficacy of new technologies need to be undertaken urgently. We must learn to work together and take the lead to help build systems that will eliminate RSI completely.
